# Pseudoaneurysm: a rare complication of distal transradial access in the anatomical snuffbox

**DOI:** 10.1186/s42155-019-0064-2

**Published:** 2019-06-29

**Authors:** Maryam Boumezrag, Bianca Ummat, Jonathan Reiner, Anthony Venbrux, Shawn Sarin

**Affiliations:** 1 Department of Radiology, George Washington University, 900 23rd St NW, Washington, DC, 20037 USA; 2 Department of Medicine, George Washington University, 900 23rd St NW, Washington, DC, 20037 USA

## Abstract

**Background:**

This report presents a case of distal radial artery pseudoaneurysm following cardiac catheterization and its successful endovascular management. Due to its novelty as a catheterization site, few to no reports exist regarding the complications associated with distal radial access.

**Case presentation:**

A patient presented to the emergency department with severe wrist and hand swelling 48 h after successful cardiac catheterization via distal radial artery access. Angiography revealed a pseudoaneurysm which was embolized with Onyx™. Post intervention angiogram showed exclusion of the pseudoaneurysm and preservation of the left palmar arch vasculature.

**Conclusion:**

The case presented herein demonstrates a rare complication of distal radial access at the anatomical snuffbox.

## Background

Transradial access has gained popularity over the femoral approach as the access site of choice, especially for percutaneous coronary interventions (PCI). The RIVAL study showed that compared to femoral access, radial access has significantly less risk of hematoma and pseudoaneurysm formation (Jolly et al. 2019). Despite these advantages, the transradial approach has drawbacks including radial artery occlusion (RAO) and nonocclusive radial inury (Sinha et al. 2017)Body habitus and shoulder mobility can also limit utilization of left radial access (Davies and Gilchrist 2018). A more recent method involving access at the anatomical snuffbox has been developed for cases in which more distal access is desired. This technique, referred to as left distal transradial angiography (ldTRA), accesses the artery at the anatomical snuffbox and can be used as an appropriate alternative to right or left radial angiography and interventions (Kiemeneij 2017). Due to its novelty as a catheterization site, only few reports exist regarding the complications associated with distal radial access at the anatomical snuffbox. We describe a novel technique for treatment of RAP using Onyx™ as the primary embolic agent. To our knowledge, there are no reports concerning embolization of iatrogenic radial artery pseudoaneurysm with Onyx™.

## Case presentation

A patient presented for planned elective cardiac catheterization as part of standard pre-Transcatheter Aortic Valve Replacement (TAVR) workup. His past medical history was significant for CAD status post CABG and mechanical mitral valve, CVA while sub-therapeutic on anticoagulation, and severe aortic stenosis. He was anticoagulated for his mechanical mitral valve and was instructed to hold Coumadin 3 days prior to the procedure. The procedure was performed via the distal left radial with a 6F JL4 catheter. Cardiac catheterization was uneventful. Due to risk of CVA risk with a mechanical valve, the patient was discharged later in the day with instructions to take Coumadin 10 mg the night of the procedure and resume Lovenox and Coumadin the following day. However, he noted progressive wrist and hand swelling over the next 48 h and was instructed to come to the emergency department.

In the ED, physical exam showed massive swelling of the left digits and the dorsal and palmar left hand, and minor swelling of the left wrist. Significant ecchymosis was noted on both surfaces of the hand. Sensation to light touch was intact. Movement was limited due to swelling and pain. Pulse oximetry in all 5 digits was > 95%. The vascular and plastic surgery services were consulted. It was not felt that fasciotomy or surgical repair was required urgently. However, despite holding manual pressure on the hand, continued hematoma formation occurred.

**Fig. 1:**
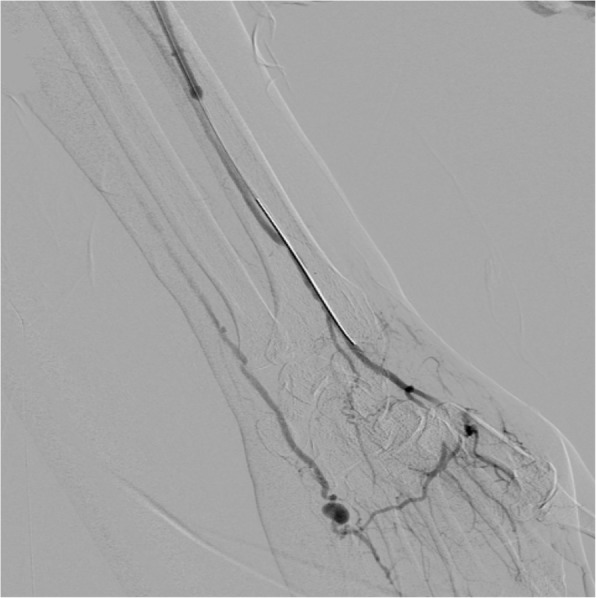
Pre-embolization angiogram showing a pseudoaneurysm in the distal aspect of the radial artery at the level of the base of the first metacarpal

**Fig. 2:**
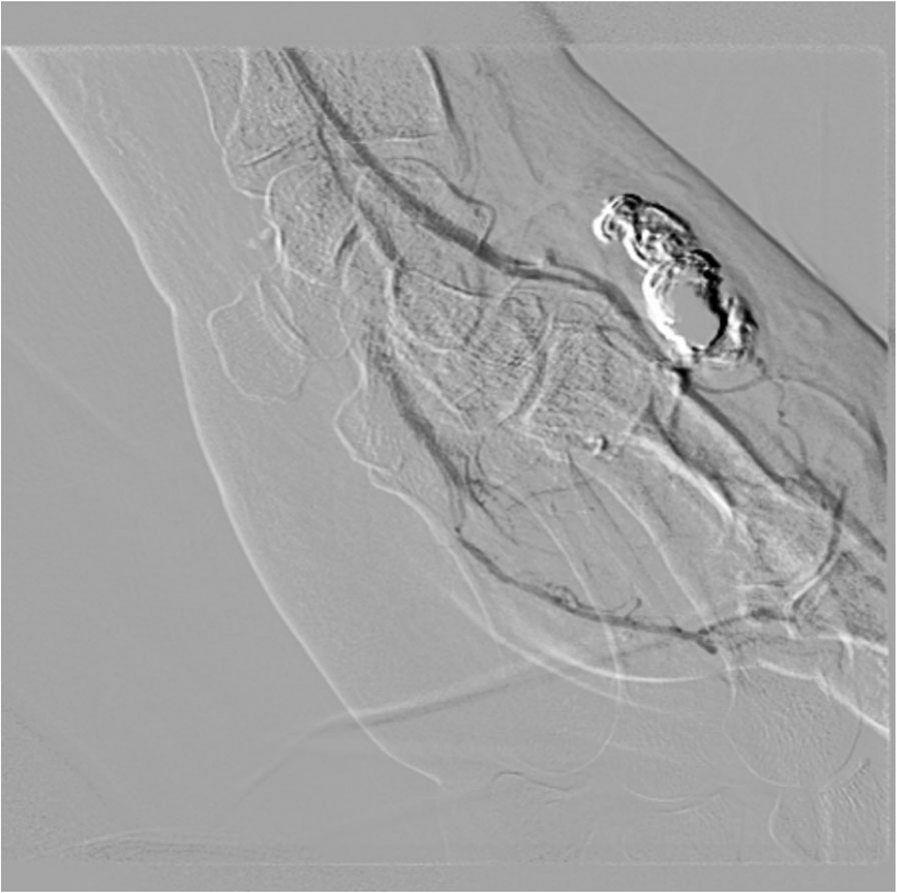
Post intervention angiogram showing exclusion of the pseudoaneurysm and preservation of the left palmar arch vasculature

The patient was brought to IR for evaluation. Access was obtained via right femoral artery. Catheter and wire were advanced under fluoroscopic guidance into the left upper extremity. There was difficulty advancing the wire and catheter combination into the left upper arm due to significant atherosclerotic disease, especially in the right common iliac artery. A multistation angiogram of the left upper extremity was performed. This showed a pseudoaneurysm off the distal aspect of the left radial artery. Advancement of a microcatheter was attempted from the right femoral access, however, was unable to advance the microcatheter beyond the level of the elbow due to significant vessel tortuosity. Therefore, it was decided to re-access the arterial system more proximal to the area of vessel injury. Using ultrasound guidance and a micropuncture set, the proximal aspect of the left radial artery was accessed in an antegrade fashion. Through the micro puncture sheath, a microcatheter was advanced into the radial artery and a selective left radial angiography performed. This confirmed presence of a pseudoaneurysm off the distal aspect of the radial artery at the level of the base of the first metacarpal (Fig. 1). The microcatheter was advanced to the level of the injury and into the pseudoaneurysm itself. Embolization material, Ethylene-Vinyl Alcohol Copolymer liquid embolic (Onyx™ 18, Medtronic, Irvine, CA), (2 cc) was administered from the catheter after first flushing the catheter with DMSO. Post intervention angiogram showed exclusion of the pseudoaneurysm and preservation of the left palmar arch vasculature (Fig. 2). Hemostasis in the right femoral access was obtained using an Angio-Seal. Hemostasis in the left radial access was obtained using a radial compression band.

Immediately following this procedure, the hand was noted to be increasingly tense and swollen with worsening skin blistering and weeping, likely related to the hand not being elevated as well as injection of contrast in to the area. The hand movement was significantly more restricted due to the swelling and pain. Sensation was intact. Patient continued to deny paresthesia. Thumb pulse ox was 95%. Radial pulse 2+ right femoral pulse 2+. The patient was monitored in the intensive care unit (ICU) for frequent neuro vascular checks. Overnight, there was improvement in the pain and swelling, which over the next day improved with elevation. The patient recovered uneventfully with no ischemic complications. Two weeks following the radial artery pseudoaneurysm repair, the patient was seen in clinic by cardiothoracic surgery for continued management of his aortic stenosis. At the time of visit, he complained of right groin pain concerning for pseudoaneurysm. An ultrasound was obtained the following day and showed a 4.7 × 4.2 × 2.4 cm hematoma but no signs of pseudoaneurysm. He was hemodynamically stable at this time and conservative management was deemed appropriate. The patient was seen again in clinic the following day and reported an improvement in his pain over 24 h. At 6 month follow-up, the patient remained asymptomatic.

## Discussion

Pseudoaneurysms are a well-known iatrogenic complication of arterial catheterization (Esposito et al. 2006). The pathogenesis originates from inadequate thrombus formation after the catheter or sheath is removed, thus causing a hematoma between the tunica media and tunica adventitia which communicates with the arterial lumen. It is estimated that pseudoaneurysms occur in .2% to 3% of femoral artery catheterizations (Stone et al. 2014). The incidence is much more rare for radial artery catheterization and is estimated at 0.009% (Tatli et al. 2015). The prevalence of radial artery pseudoaneurysm (RAP) is likely to increase in the future as radial access continues to grow in popularity.

The diagnosis of RAP is usually based on a high degree of clinical suspicion and often followed by a doppler ultrasound. Several other imaging modalities can also be used, though angiography remains the gold standard for diagnosis (Mohan et al. 2017).

The potential risks associated with RAP can be significant due to the artery’s proximity to the palmar arch as well as its superficial location within a highly mobile region of the wrist. These risks include distal thromboembolism, digital ischemia, hemarthrosis, parasthesias, rupture, skin ulceration, and secondary infection thus prompting timely intervention (Luzzani et al. 2006; Gabriel et al. 2008). Though no report exist to date, these risks may be higher in cases such as the one described in which the radial artery pseudoaneurysm is located at the anatomical snuffbox.

Several treatment options exist in managing RAP including compression management, ultrasound-guided thrombin injection, and surgical excision with or without radial artery ligation (Nassiri et al. 2016).

While it is the least invasive option, ultrasound-guided compression therapy has a high failure rate and usually requires for the pseudoaneurysm to be located superficially within muscle compartments. (Mohan et al. 2017; Eisenberg et al. 1999). This method is even less successful in patient on anticoagulation (Chandradev and Ateesh 2014).

Similarly, percutaneous thrombin injection can be severely limited by pseudoaneurysm characteristics, namely pseudoaneurysm base morphology and neck length In cases in which the pseudoaneurysm has a broad base and a short neck, there is a high risk of distal digital ischemia from non-target embolization. Thus, assessing a patient’s candidacy for percutaneous thrombin injection requires careful evaluation and scrutiny of the pseudoaneurysm morphology on duplex ultrasonography (Nassiri et al. 2016). Finally, surgery is the most invasive option and may not be an option for patients with multiple comorbidities such as the one described.

In the case described, the embolic agent of choice was ONYX™ liquid embolic system (Medtronic, Irvine, CA) (Ling et al. 2006). To our knowledge, no cases of Onyx™ injection for the treatment of radial artery pseudoaneurysms have been published. Onyx™ is becoming more widespread among interventional radiologists in the treatment of peripheral lesions. It has been used for several years in the treatment of arteriovenous malformations (AVMs) and more recently for filling endoleaks and in peripheral AVMs (Kolber et al. 2015; Guimaraes and Wooster 2011; Urbano et al. 2014; Numan et al. 2004; Abularrage et al. 2011). The material is comprised of EVOH (ethylene vinyl alcohol) copolymer dissolved in DMSO (dimethyl sulfoxide), and suspended micronized tantalum powder to provide contrast for visualization under fluoroscopy. It is available in two formulations: Onyx®-18 (6% EVOH), which was used in the case described, and Onyx®-34 (8% EVOH).

Several characteristics of Onyx allow it to achieve controlled, deliberate, and predictable thrombosis. Onyx has a high viscosity and long polymerization time which allows for slow administration. Unlike glue, Onyx only precipitates in the absence of DMSO. Therefore, preloading microcatheters with DMSO prevents cementing of the catheter tip. This allows the operator to pause and resume injection while leaving the catheter in place. Once it comes in contact with blood, Onyx forms a soft spongy polymer cast. This process begins at the surface while the core remains liquid. This quality of the material creates a smooth flow pattern without any fragmentation during injection and reduces the risk of non-target embolization (Weber et al. 2007).

## Conclusion

We report a case of successful transarterial embolization of a radial artery pseudoaneurysm following cardiac catheterization at the anatomical snuffbox. Onyx™ was successfully injected, excluding the pseudoaneurysm.

## Data Availability

Data sharing not applicable to this article as no datasets were generated or analyzed during the current study.

## Abbreviations

DMSO: Dimethyl sulfoxide
ldTRA: Left distal transradial angiography
PCI: Percutaneous coronary intervention
RAO: Radial artery occlusion
TAVR: Transcatheter Aortic Valve Replacement
